# Antioxidant Activities of *Vaccinium vitis-idaea* L. Leaves within Cultivars and Their Phenolic Compounds

**DOI:** 10.3390/molecules24050844

**Published:** 2019-02-27

**Authors:** Lina Raudone, Gabriele Vilkickyte, Lina Pitkauskaite, Raimondas Raudonis, Rimanta Vainoriene, Vida Motiekaityte

**Affiliations:** 1 Department of Pharmacognosy, Lithuanian University of Health Sciences, Sukileliu av. 13, LT-50162 Kaunas, Lithuania; gabriele.vilkickyte@fc.lsmuni.lt (G.V.); lina.pitkauskaite@fc.lsmuni.lt (L.P.); raimondas.raudonis@lsmuni.lt (R.R.); 2 Laboratory of Biopharmaceutical Research, Institute of Pharmaceutical Technologies, Lithuanian University of Health Sciences, Sukileliu av. 13, LT-50162 Kaunas, Lithuania; 3 The Botanical Garden of Siauliai University, Paitaiciu str. 4, LT-77175 Siauliai, Lithuania; rimanta.vainoriene@su.lt; 4 Biomedical Sciences Department, Siauliai State College. Ausros av. 40, LT-76241 Siauliai, Lithuania; vmotiek@gmail.com

**Keywords:** lingonberry, *Vaccinium vitis-idaea*, antioxidant activity, phenolic compounds, HPLC–PDA, structure-activity relationship

## Abstract

Lingonberry leaves are the subject of numerous studies because of antioxidant properties, positive influence on the health and potential use in the prevention and treatment of chronic diseases. In this work, the radical scavenging, reducing, chelating activities, and phenolic composition of ten lingonberry leaves cultivars, one subspecies, and one variety were investigated. Furthermore, the antioxidant activity of individual phenolic compounds, that can be found in lingonberry leaves, were analyzed, and structure-activity relationship was determined. Wide diversity for phenolic profile and antioxidant properties of lingonberry leaves has been observed in the present material. Cultivars ‘Kostromskaja rozovaja’, ‘Rubin’, and *Vaccinium vitis-idaea* var. *leucocarpum* surpassed all others tested cultivars and lower taxa by contents of phenolic compounds and antioxidant activity. Leaves of lingonberry cultivars and lower taxa are rich in arbutin, flavonol glycosides, proanthocyanidins, and the latter were considered to be the major contributor to antioxidant properties of lingonberry leaves.

## 1. Introduction

Lingonberry (*Vaccinium vitis-idaea* L.) is an evergreen shrub of the *Ericaceae* Juss. family. It is one of the most popular berries in Nordic countries, as well as in Baltic States, Russia and Canada [1,2]. Lingonberry raw materials are mainly collected in natural habitats. In Lithuania the area and productivity of the natural populations of lingonberries are declining, as well as they are unevenly distributed [3]. Research on natural resources assessment is relevant because anthropogenic factors have a negative impact on the populations of lingonberry. Sustainable use of natural resources becomes of crucial importance. Cultivation of introduced and locally selected lingonberries could be the best option to provide for the increasing needs of this plant material. Lingonberries are widely used in the human diet due to nutritional benefits, moreover, leaves of this plant can be used for prevention and treatment of urinary tract infections, stomach disorders, rheumatic diseases and hypercholesterolemia [1,2,3,4,5]. The wide spectrum of various biological properties of lingonberry leaves, including antioxidant activity is related to their phenolic constituents [4,6,7].

It is known that phenolic compounds can transfer an electron or hydrogen atom from the phenolic group to the reactive free radicals, and thus act as reducing agents or free radical scavengers. Also, they are able to form σ bonds, and therefore to chelate transition metals, which are powerful pro-oxidants. Because of several mechanisms of action, phenolic compounds ensure the effective defense mechanism against potentially harmful radical species, hence predispose preventing or postponing the onset of various human degenerative and chronic diseases [8,9,10]. Differences between the predominant mechanism of action and antioxidant properties of phenolic compounds are due to the number and positions of hydroxyl groups, the sugar moiety and other features in the chemical structure. Structural diversity also determines the differences between reactivity and reaction kinetics of phenolic compounds [8,11,12]. For some compounds (i.e., *p*-coumaric acid, gallic acid, caffeic acid, ferulic acid, (+)-catechin, gallocatechin, catechin gallate, gallocatechin gallate, rutin) there is no or small initial fast reaction and slow continuous reaction afterward [13]. In the online post-column methods, the reaction in the reactor between antioxidant and reagent usually lasts for less than a minute. Consequently, the antioxidant activity of the slow-acting compounds could be evaluated inaccurately. Therefore, antioxidant activities of phenolic compounds, which can be detected in lingonberry leaves, were evaluated by spectrophotometric methods of analysis in the end point of reaction.

Many factors are related to the production of phenolic compounds in plants, among this, there are external factors, such as exposure of the light, solar radiation, temperature, type of soil, cultivation methods, insect attacks, and internal factors—genetic background. These factors especially genetic diversity, cause wide variations even within a single species [14,15]. Therefore we have chosen to analyze ten different cultivars, one subspecies and one variety of lingonberries. To the best of our knowledge, the antioxidant activity and phenolic composition of these certain cultivars and lower taxa of lingonberries—‘Sussi’, ‘Erntekrone’, ‘Masovia’, ‘Koralle’, ‘Kostromskaja rozovaja’, ‘Sanna’, ‘Rubin’, ‘Kostromička’, ‘Erntesegen’, ‘Erntedank’, *V. vitis-idaea* subsp. *minus*, and *V. vitis-idaea* var. *leucocarpum* have not been compared and determined before.

This study was carried out to determine the radical scavenging, reducing and chelating activities of different cultivars and lower taxa of lingonberry leaves extracts, also to evaluate phenolics composition and major contributors to the antioxidant properties. In addition, the structure-antioxidant activity relationship of phenolic compounds detected in lingonberry leaves were analyzed.

## 2. Results

### 2.1. Antioxidant Activity of Lingonberry Leaves Extracts

#### 2.1.1. ABTS Radical Scavenging Activity

**Figure 1:**
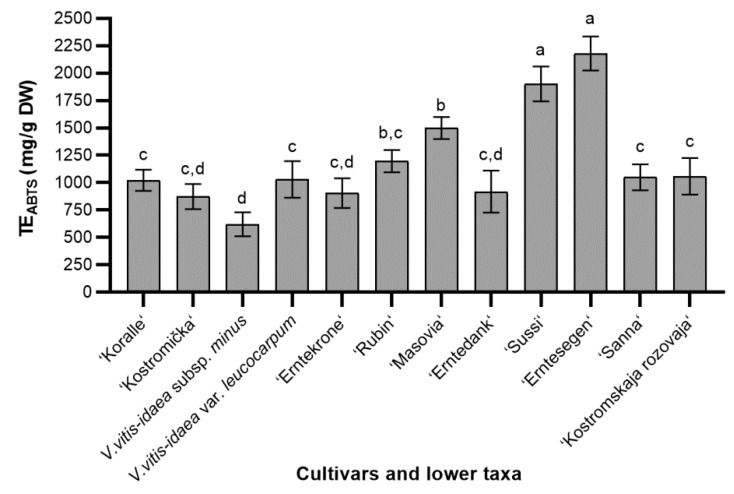
The radical scavenging activity of different cultivars and lower taxa of lingonberry leaves extracts; bars without the same letters (a, b, c, d) indicate statistically significant differences between the means (*p* < 0.05).

The radical scavenging activity of different lingonberry cultivars and lower taxa is shown in Figure 1. The radical scavenging activity ranged from 620.49 to 2179.71 mg/g dry weight (DW). The average of radical scavenging activity of different cultivars and lower taxa was 1188.04 mg/g DW. The obtained results clearly showed that the highest radical scavenging activity (2179.71 ± 154.88 mg/g DW) was determined in ‘Erntesegen’ cultivar—almost two times more than the average. There were no significant differences between ‘Erntesegen’ and ‘Sussi’ cultivars. Radical scavenging activity of ‘Rubin’ and ‘Kostromskaja rozovaja’ cultivars was a bit lower than average, whereas other antioxidant activity assays demonstrated that these cultivars showed strong antioxidant activity. The lowest radical scavenging activity was found in *V. vitis-idaea* subsp. *minus*, ‘Kostromička’, ‘Erntekrone’, and ‘Erntedank’ cultivars.

#### 2.1.2. Ferric Reducing Antioxidant Power (FRAP)

**Figure 2:**
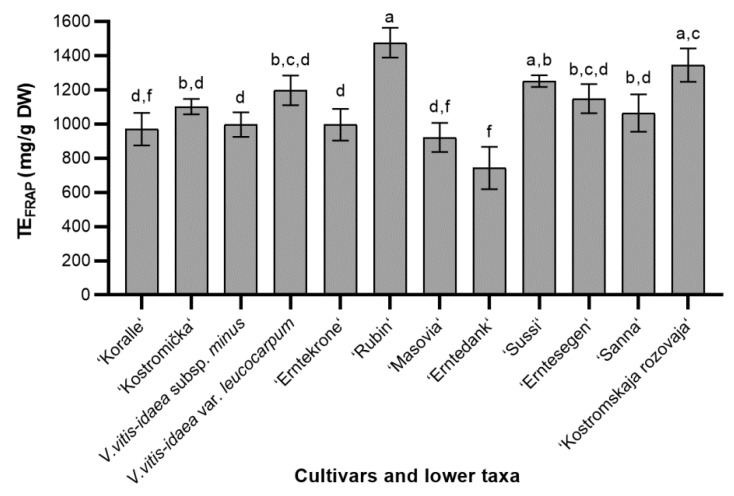
The reducing activity of different cultivars and lower taxa of lingonberry leaves extracts; bars without the same letters (a, b, c, d, f) indicate statistically significant differences between the means (*p* < 0.05).

The results provided in Figure 2 demonstrate the reducing activity of the different lingonberry cultivars and lower taxa. The reducing activity among the cultivars and lower taxa studied ranged with values from 743.38 to 1476.04 mg/g DW. The average of reducing power activity of different cultivars and lower taxa was 1101.32 mg/g DW. ‘Rubin’, ‘Sussi’ and ‘Kostromskaja rozovaja’ presented the highest reducing power activity and they did not differ significantly. These cultivars demonstrated significantly strong antioxidant activity compared to ‘Erntedank’ and ‘Masovia’ cultivars. Higher than average reducing activity was determined in *V. vitis-idaea* var. *leucocarpum* and ‘Erntesegen’ cultivar. Overall, ‘Rubin’ leaves revealed the greatest antioxidant properties, whereas the ‘Erntedank’ leaves possessed a very poor antioxidant activity—almost one and a half times lower than average.

#### 2.1.3. Ferrous Ions Chelating (FIC) Activity

**Figure 3:**
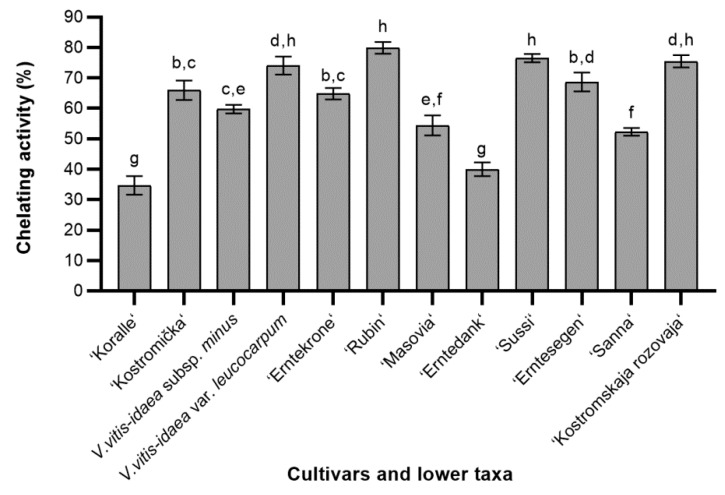
The chelating activity of different cultivars and lower taxa of lingonberry leaves extracts; bars without the same letters (a, b, c, d, e, f, g, h) indicate statistically significant differences between the means (*p* < 0.05).

Chelating activity showed statistically significant variations throughout the different cultivars and lower taxa of lingonberry leave extracts (Figure 3). The ability of extracts to chelate ferrous ions ranged between 34.67 ± 3.05% and 79.85 ± 1.92%. The average of chelating activity of different cultivars and lower taxa of lingonberries was 62.21%. In this study, the highest chelating activity was determined in ‘Rubin’ cultivar, and it was almost the same as 10 µg/mL of standard chelator—EDTA (data not shown). There were no significant differences between the *V. vitis-idaea* var. *leucocarpum* and ‘Rubin’, ‘Sussi’, ‘Kostromskaja rozovaja’ cultivars. Higher than average chelating ability was also established in ‘Kostromička’, ‘Erntesegen’, and ‘Entekrone’ cultivars. This suggests that these cultivars of lingonberries are rich in compounds, which captures ferrous ions before ferrozine. Meanwhile, the lowest chelating activity—almost two times lower than average, was found in ‘Koralle’ cultivar, and one and a half times lower—in ‘Erntedank’ cultivar. By comparison with other cultivars, these differences were statistically significant. Obtained results indicate that there is a wide genetic diversity for chelating properties of lingonberry leaves, and chelating activity possibly depends on the phytochemical content of cultivar or lower taxa.

### 2.2. Qualitative and Quantitative Analysis of Phenolic Compounds in Lingonberry Leaves

The results of high–performance liquid chromatography-photodiode array detection (HPLC-PDA) method of analysis, showed that eleven phenolic compounds, belonging to subgroups of simple phenols, flavonols, flavanols, proanthocyanidins, and hydroxycinnamic acids were detected in different cultivars, and lower taxa of lingonberry leaves extracts (Table 1, Table 2).

Arbutin was the most abundant phenolic compound in all tested cultivars and lower taxa (Table 1). This compound contributed approximately 41–78% of the total phenolics in different cultivars and lower taxa of lingonberries. The coefficient of variation (CV) of arbutin among tested cultivars and lower taxa was 67%. Significantly the highest content of arbutin was found in ‘Kostromskaja rozovaja’, followed by ‘Rubin’ cultivar, meanwhile the lowest in ‘Sanna’, ‘Erntedank’, ‘Erntesegen’ cultivars of lingonberries.

Large amounts of some flavonols in the present study were found (Table 1). Significantly the highest contents of avicularin were determined in *V. vitis-idaea* var. *leucocarpum*, whereas that of hyperoside were found in *V. vitis-idaea* subsp. *minus* and *V. vitis-idaea* var. *leucocarpum*. The CV of avicularin (39%) and hyperoside (47.5%) were the lowest of all detected compounds. It was determined, that quercitrin was predominant flavonol in *V. vitis-idaea* subsp. *minus* and ‘Sussi’, ‘Koralle’, ‘Sanna’, and ‘Erntedank’ cultivars of lingonberries. Amounts of astragalin were considerably lower than that of other phenolic compounds, and ranged between 3.07 ± 0.13 µg/g DW in ‘Masovia’ and 120.60 ± 4.92 µg/g DW in ‘Rubin’ cultivar. The sum of flavonol glycosides made up from 9% to 41% of the phenolic content in ‘Kostromskaja rozovaja’ and ‘Koralle’ cultivars, respectively.

The determined amounts of (+)-catechin and (−)-epicatechin varied within a very wide range (Table 2). The CV of these compounds was 91% and 129%, respectively. The peak of (−)-epicatechin was not detected in ‘Erntesegen’ and ‘Erntedank’ cultivars. Amounts of monomeric flavanols, mentioned above, were lower than those of oligomeric proanthocyanidins. The content of procyanidin C1 among the tested cultivars and lower taxa ranged between 936.48 ± 38.23 and 4645.97 ± 189.67 µg/g DW with the highest amount determined in *V. vitis-idaea* var. *leucocarpum*. Whereas content of procyanidin A2 varied from 476.93 ± 19.47 to 7074.60 ± 288.82 µg/g DW with the highest amount in *V. vitis-idaea* subsp. *minus*. Procyanidins C1 and A2 contribution to *V. vitis-idaea* var. *leucocarpum* either *V. vitis-idaea* subsp. *minus* was about 16–17% of the total detected phenolics. The total content of flavanols and proanthocyanidins ranged between 1584.70 ± 64.70 µg/g DW in ‘Erntedank’ cultivar and 9932.02 ± 405.47 µg/g DW in *V. vitis-idaea* var. *leucocarpum*.

The sum of detected hydroxycinnamic acids made up only 1–4% of the phenolic content in different cultivars and lower taxa of lingonberries (Table 2). The CV was 55% and 59% for chlorogenic acid and cryptochlorogenic acid, respectively. Highest contents of chlorogenic and cryptochlorogenic acids were determined in the extracts from *V. vitis-idaea* var. *leucocarpum*.

Concerning the total amounts of detected phenolic compounds within the cultivars, it was determined that the greatest amounts of compounds were in the extracts from ‘Rubin’ and ‘Kostromskaja rozovaja’ cultivars, whereas the lowest ones—in ‘Erntedank’, ‘Erntesegen’, and ‘Sanna’ cultivars (data not shown).

### 2.3. Hierarchical Cluster Analysis of Phenolic Compounds of Lingonberry Leaves of Different Cultivars and Lower Taxa

**Figure 4:**
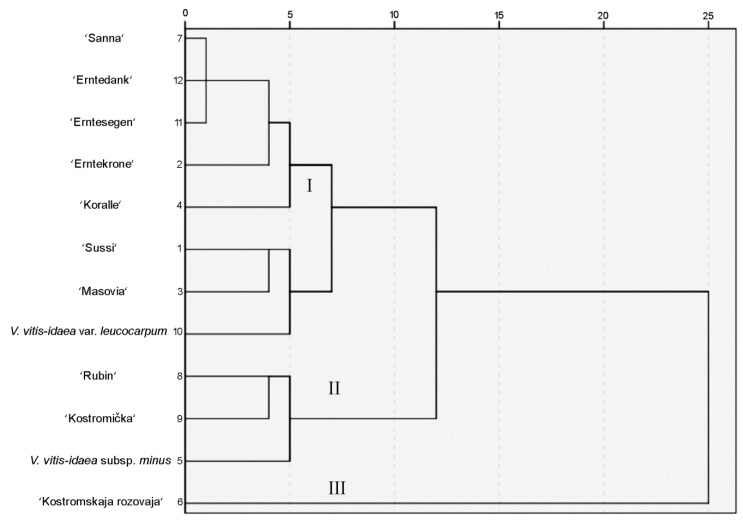
Dendrogram based on the amounts of phenolic compounds of lingonberry cultivars and lower taxa.

The cluster analysis has separated investigated lingonberry cultivars and lower taxa into three statistically significant clusters (Figure 4). The lingonberry cultivars attributed of the first cluster were German cultivars—‘Erntekrone’, ‘Erntesegen’, ‘Erntedank’, Swedish cultivars—‘Sanna’ and ‘Sussi’, ‘Koralle’, ‘Masovia’, and *V. vitis-idaea* var. *leucocarpum*. Leave samples of this cluster were characterized by lower levels of all identified compounds and significantly the lowest arbutin and procyanidins A2 and C1 levels (13,809.08 µg/g, 2232.95 µg/g, 1601.57 µg/g DW, respectively). The predominant compound and cluster marker was quercitrin (on the average 2916.17 µg/g DW). The cluster two was distinguished by the highest (*p* < 0.05) amounts of quercitrin, hyperoside and procyanidin A2 (on the average 3627.50 µg/g, 4125.29 µg/g, 4289.51 µg/g DW, respectively). Lingonberries of Russian (‘Rubin’, ‘Kostromička’) and Canada origin (*V. vitis-idaea* subsp. *minus*) were attributed to this cluster. The Russian origin ‘Kostromskaja rozovaja’ was attributed to the third cluster, which was characterized by the highest (*p* < 0.05) amounts of astragalin and procyanidin C1 (3894.92 µg/g DW) and the lowest (*p* < 0.05) amounts of (−)-epicatechin.

### 2.4. Antioxidant Activity of Lingonberries Phenolic Compounds

**Figure 5:**
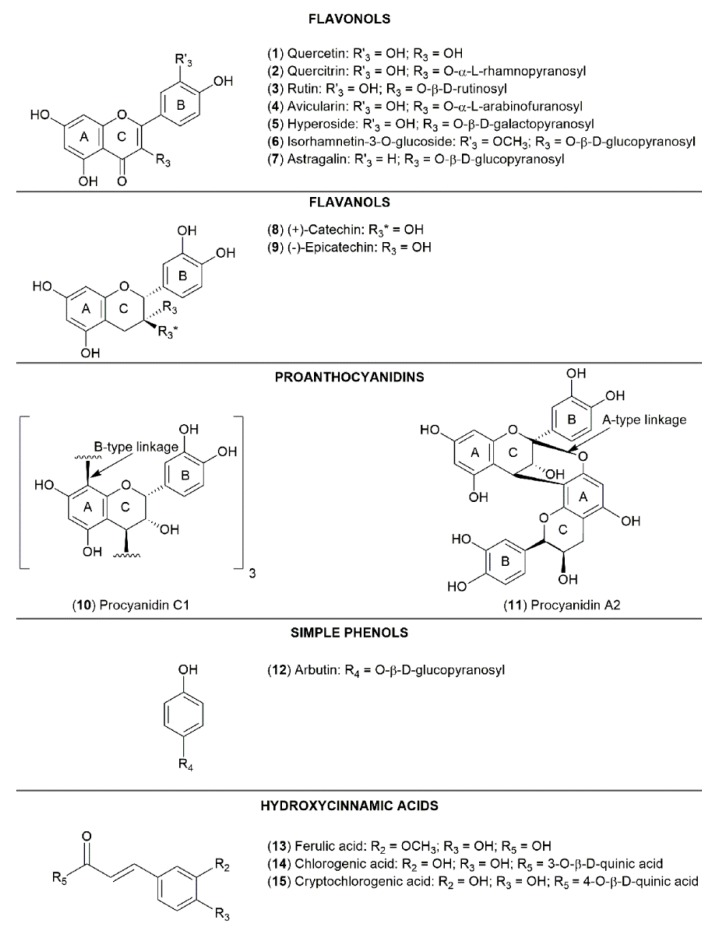
The chemical structures of studied phenolic compounds (**1**–**15**).

In purpose of better understanding of structure-antioxidant activity relationship, we investigated not only standards of phenolic compounds, which we detect in our study but also several compounds which were detected in lingonberry materials in previous studies. The chemical structures of phenolic compounds, that can be found in lingonberry leaves, and have been investigated in our study, are shown in Figure 5.

#### 2.4.1. ABTS Radical Scavenging Activity

The radical scavenging activity of tested phenolic compounds and Trolox increased with their concentration. All calibration curves equations followed a linear regression model (*p* < 0.05) (Table 3).

**Figure 6:**
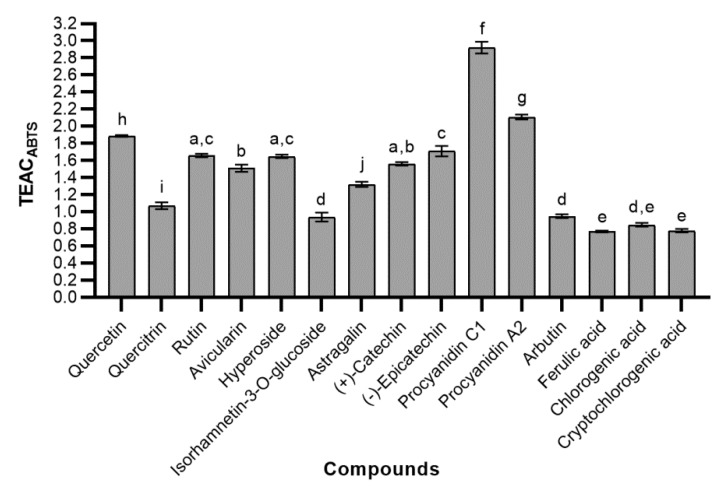
The radical scavenging activity of tested phenolic compounds; bars without the same letters (a, b, c, d, e, f, g, h, i, j) indicate statistically significant differences between the means (*p* < 0.05).

The Trolox equivalent antioxidant capacity values obtained by the ABTS assay (TEAC_ABTS_) indicated how many times the radical scavenging activity of the tested phenolic compound was greater than that of Trolox (Figure 6). Significantly the greatest TEAC_ABTS_ values were determined for proanthocyanidins—procyanidins C1 and A2. Their scavenging activity was 2.92 ± 0.07 and 2.11 ± 0.03 times greater than Trolox, respectively. TEAC_ABTS_ of monomeric flavanols was considerably lower than that of oligomeric polyphenols, mentioned above. The radical scavenging activity of (−)-epicatechin (1.71 ± 0.06) was greater than that of (+)-catechin (1.56 ± 0.02), and this difference was statistically significant. Among tested flavonols, quercetin was the most effective scavenger of the ABTS cation radical (1.89 ± 0.01). Glycosylation of the 3-hydroxyl in the quercetin molecule had an effect on the scavenging activity, as a result, TEAC_ABTS_ of quercitrin, rutin, avicularin, and hyperoside was significantly inferior to quercetin. The scavenging activity of kaempferol glycoside—astragalin was lower than that of all quercetin glycosides, with the exception of quercitrin. Alkylation of catechol moiety further reduced ability to scavenge radicals, therefore TEAC_ABTS_ of isorhamnetin-3-glucoside was lowest of all tested flavonoids (0.94 ± 0.05). Its TEAC_ABTS_ was almost the same as that of glycosylated hydroquinone—arbutin (0.95 ± 0.02). The results showed, that hydroxycinnamic acids (ferulic, chlorogenic, and cryptochlorogenic acids) were less active than tested flavonoids, and despite structural differences, there was no statistically significant disparity among them.

#### 2.4.2. Ferric Reducing Antioxidant Power (FRAP)

It was determined that the reducing power of tested phenolic compounds and Trolox was concentration dependent. All calibration curves equations followed a linear regression model (*p* < 0.05) (Table 4).

**Figure 7:**
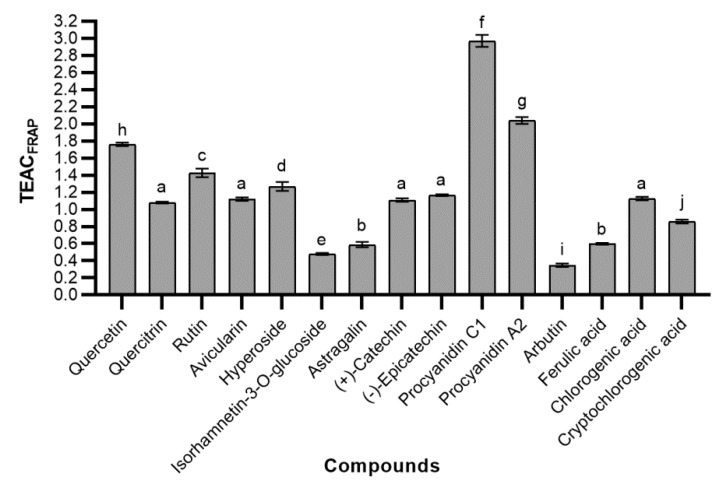
The reducing activity of tested phenolic compounds; bars without the same letters (a, b, c, d, e, f, g, h, i, j) indicate statistically significant differences between the means (*p* < 0.05).

The Trolox equivalent antioxidant capacity values obtained by the FRAP assay (TEAC_FRAP_) revealed how many times the reducing activity of the tested phenolic compound was greater than that of Trolox (Figure 7). The profile of reducing activity was similar to the results of the free radical scavenging activity. As in the ABTS assay, proanthocyanidins showed the greatest activity. TEAC_FRAP_ was 2.97 ± 0.07 and 2.04 ± 0.04 for procyanidins C1 and A2, respectively. These results demonstrate that proanthocyanidins have a strong potency to donate electrons to reactive free radicals, converting them into more stable forms. In this assay there was no statistically significant difference in reducing activity between different isomers of catechin. As expected, quercetin was the most active flavonoid (1.76 ± 0.02). High TEAC_FRAP_ was also observed in some quercetin glycosides—rutin (1.43 ± 0.05) and hyperoside (1.27 ± 0.05). The lowest reducing power had arbutin—its ability to reduce ferric ions was about three times lower than Trolox, and even eight and a half times lower than procyanidin C1. It was assessed that TEAC_FRAP_ was 0.60 ± 0.01, 1.13 ± 0.02, and 0,86 ± 0.02 for ferulic acid, chlorogenic acid, and cryptochlorogenic acid, respectively.

#### 2.4.3. Ferrous Ions Chelating (FIC) Activity

**Figure 8:**
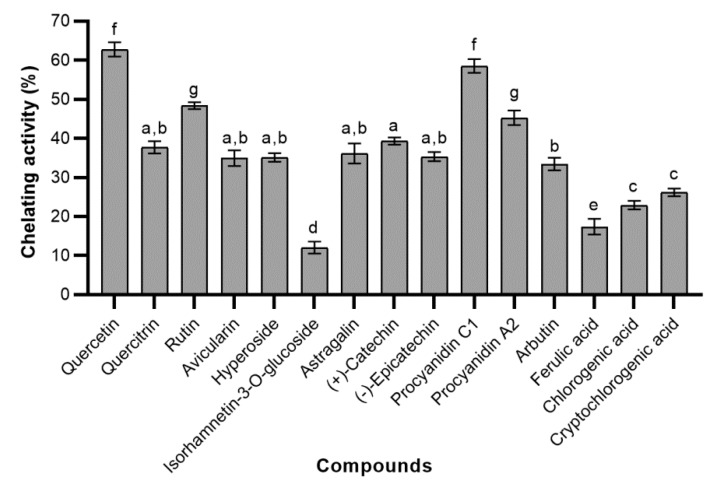
The chelating activity of tested phenolic compounds at a concentration of 20 μg/mL; bars without the same letters (a, b, c, d, e, f, g) indicate statistically significant differences between the means (*p* < 0.05).

In the ferrous ions chelating assay, we did not obtain the linear concentration-response curves, because results of simple linear regression analysis showed that there was no linear relationship between concentrations of phenolic compounds (tested concentration range 5–80 µg/mL) and chelating activity (*p* > 0.05 for regression coefficients). Therefore we only investigated and compared the chelating activity of the same 20 µg/mL concentration of the standards of phenolic compounds. All tested phenolic compounds were able to chelate ferrous ions at this concentration, nevertheless, they possessed significantly lower chelating activity than the same concentration chelating agent—EDTA (88.55 ± 2.84%). The greatest ability to chelate ferrous ions was observed in quercetin followed by procyanidin C1 (62.74 ± 1.80% and 58.53 ± 1.73%, respectively) (Figure 8). Rutin was the most active quercetin glycoside, its chelating activity was similar to procyanidin A2 (48.38 ± 0.91% and 45.25 ± 1.86%, respectively). It was determined, that the type of other glycosides had no significant influence on the chelating activity. Furthermore, the chelating activity of all quercetin glycosides, except rutin, did not differ significantly from kaempferol glycoside and flavanols. The highest influence on chelating activity had methylation of catechol moiety, hence isorhamnetin-3-*O*-glucoside had the lowest activity of all tested compounds (12.08 ± 1.55%). Surprisingly, high chelating activity was determined in arbutin (33.44 ± 1.63%). We hypothesize that this is likely due to the specific pharmacophore and differences in reaction stoichiometry. By comparing the chelating activity of phenolic acids, a similar trend as in FRAP assay was found, with the exception, that there was no difference between the activity of chlorogenic acid isomers.

### 2.5. Correlation Analysis

Antioxidant activities of lingonberry leaves extracts were correlated to the amounts of their phenolic constituents. There was a moderate positive correlation between reducing activity and the contents of cryptochlorogenic acid (R = 0.609, *p* < 0.05), (−)-epicatechin (R = 0.593, *p* < 0.05), arbutin (R = 0.622, *p* < 0.05), and furthermore between chelating activity and the contents of cryptochlorogenic acid (R = 0.665, *p* < 0.05) and arbutin (R = 0.621, *p* < 0.05). No significant correlations were determined between antioxidant activity and contents of other tested compounds.

Correlations between methods of analysis measuring different antioxidant properties were also investigated. There was an overall strong positive correlation between FIC and FRAP assays measuring antioxidant properties in cultivars of lingonberry leaves extracts (R = 0.844, *p* < 0.05). The assessed TEAC_ABTS_ of studied phenolic compounds were similar to TEAC_FRAP_ (R = 0.904, *p* < 0.05). Moreover, there were a strong correlations between FIC values and TEAC_ABTS_ (R = 0.817, *p* < 0.05) either TEAC_FRAP_ (R = 0.766, *p* < 0.05). This indicates that there is a relationship between the mechanism of action of phenolic compounds, and the same structural fragments may be responsible for different antioxidant properties.

## 3. Discussion

Phytochemical composition of lingonberry leaves strongly depends on the growing region. In our study, we have determined the quantitative and qualitative composition of bioactive compounds in lingonberries, the predominant compounds in phytochemical and antioxidant profiles, the differences in bioactive compound complexes of the distinct cultivated cultivars, subspecies, and variety.

Antioxidant properties of lingonberry leaves are in agreement with previous studies. Bujor et al. compared antioxidant activity between different parts of lingonberry, and determined that the antioxidant activity of lingonberry leaves is considerably greater than that of fruits [4]. Enkhtuya et al., investigated radical scavenging and reducing activities of different plants, and found that in DPPH, ABTS, and FRAP methods of analysis lingonberry leaves were more active than leaves of black currant, hawthorn, strawberry, and tall currant. Radical scavenging and reducing properties of lingonberry leaves were similar to that of seabuckthorn [16]. Vyas et al. found significant differences of radical scavenging and reducing activities of lingonberry leaves within wild clones and cultivars, and distinguished *V. vitis-idaea* subsp. *minus* with high antioxidant properties [7]. Tian et al. assessed that within 10 min the lingonberry leaves extracts scavenged 86.3 ± 2.5% of DPPH radicals, and had an extremely high ORAC activity [13]. This indicates that lingonberry leaves exhibit great ability to donate hydrogen atom or transfer single–electron. Concerning chelating activity, some researchers reported that lingonberry extracts showed weaker chelating activity than extracts from plants belonging to other families [16,17]. However, it is difficult to compare the antioxidant activity results obtained in the present study with the literature data, because of the differences in methods and expression of antioxidant activity.

Based on our findings of the qualitative and quantitative analysis, arbutin was the predominant phenolic compound in all cultivars and lower taxa ranging from 7070.25 ± 288.64 µg/g up to 56,968.56 ± 2325.73 µg/g DW. Arbutin has been reported in previous studies to be a major phenolic constituent of lingonberry leaves [18,19]. The HPLC–PDA study of arbutin content in lingonberry leaves has given the result of 5168 ± 480 µg/g DW [20], which is similar to the result obtained in ‘Kostromskaja rozovaja’ cultivar.

Large amounts of flavonols in lingonberries have been confirmed by several researchers. Liu et al. determined that flavonol glycosides were the second major group of phenolics in lingonberry leaves [1]. Tian et al. emphasized that amount of flavonol glycosides is one of the main difference of phenolic profile between lingonberry leaves and fruits and assessed that contents of flavonol glycosides are considerably greater in lingonberry leaves [21]. Scientific studies demonstrate that not only hyperoside, avicularin, quercitrin, and astragalin can be detected in lingonberry leaves but also quercetin aglycon, rutin, and 3-*O*-glycosides of quercetin, isorhamnetin, and kaempferol [18,19,21,22,23].

(+)-Catechin, (−)-epicatechin, proanthocyanidins, primarily as A-type or B-type procyanidin dimers and trimers, have been reported in a number of publications to be widespread in lingonberry leaves [1,13,23,24,25,26]. However, the determination of proanthocyanidins by HPLC is problematic because proanthocyanidins often occur in complexes, that makes their separation and detection difficult [22]. This suggests that there are not only procyanidins C1 and A2, which were detected in present material but also more types of proanthocyanidins.

Low contents of hydroxycinnamic acids obtained in our research were partly consistent with previous studies. Bujor et al. reported that hydroxycinnamic acids represent the less abundant group of phenolic compounds in the lingonberry leaves. However, they found higher levels of hydroxycinnamic acids, because the relative content of hydroxycinnamic acids and their derivatives was in the range of 6–14% in leaves [4]. The presence of caffeoylquinic acid isomers is in agreement with their detection in lingonberries in various studies [18,23]. Nevertheless, Tian et al. disclosed that caffeoylquinic acids were not predominant compounds in lingonberry extract [13]. Other phenolic acids in various studies were detected in the extracts of lingonberries, i.e., coumaroyl quinic acid isomers (3.81% of the total combined peak area of all compounds), p-coumaric acid (0.64%), caffeic acid (0.61%), caffeoyl-shikimic acid (0.18%), feruloyl quinic acid (0.04%) [22], and also hexoses of ferulic acid [13], and free ferulic acid [27].

After the analysis of phytochemical and antioxidant profiles of separate lingonberry cultivars and lower taxa, there were no determined relationships with the country of origin of lingonberries nor the conditions of plants growing in trial areas. The cluster analysis revealed that according to the composition of phenolic compounds, the clusters were related to the countries of origin, especially with German and Russian origin cultivars. The predominant phenolic markers in German cultivars (‘Erntekrone’, ‘Erntesegen’, and ‘Erntedank’) were hyperoside and quercitrin, while in the Russian cultivars—procyanidins A2 and C1. In the study of phenolic compounds, it has been shown that in plant phylogeny and in the systematics, the composition of aglycons is a conservative feature that is very little dependent on the conditions of growth and denotes the specificity of genus and lower taxa [28]. Based on this research mentioned above, the composition of the phenolic compounds and their dominant markers are currently applied in both plant genotype studies [29], as well as antioxidant activity studies of these tribal representatives [28,30].

According to the results of radical scavenging, reducing and chelating activities of Lingonberries phenolic compounds, proanthocyanidins, which include in their structure epicatechin units, can be characterized by exceptionally strong antioxidant properties. These results agreed with those of Muselík et al. [31] and Spranger et al. [32], who reported that proanthocyanidins have the highest antioxidant activity among all natural phenolic compounds. This may be due to more hydroxyl groups, which act as strong hydrogen donors, generating by itself stable intramolecular hydrogen bonds with semi-quinoid free radicals and o–quinines, and thus disrupt radical chain reaction [33]. It was also found that B-type trimeric proanthocyanidins possess greater antioxidant activity than A-type dimeric proanthocyanidins.

In our study, all phenolic glycosides had lower antioxidant activity than their corresponding aglycons. It confirms that *O*-glycosylation reduces the antioxidant activity of phenolic compounds [11,34,35]. It is believed that 3-*O*-glycosylation increases torsion angle and interferes the planarity of rings, leading to suppression of antioxidant activity [21]. Furthermore, it was noticed that in present study substitution of methoxyl group had a strong impact for reducing and chelating activities, whereas had a weaker impact for the radical scavenging activity. This suggests that the presence of electron donating methoxyl group adjacent to the hydroxyl group alters the redox potential and might enhance the hydrogen availability, donating capacity and thus radical scavenging activity [36,37]. Grzesik et al. discovered that the scavenging activity of the ABTS cation radical of ferulic acid was even greater than that of chlorogenic acid, meanwhile ability to reduce ferric ions was weaker [38].

The importance of catechol moiety (*ortho*–dihydroxyl structure) in the ring B was emphasized by previous studies [9,12,34]. In the present study, it was observed that catechol moiety increases the antioxidant activity because all quercetin glycosides still showed higher radical scavenging and reducing activities than kaempferol glycoside. Moreover, in all methods of analysis, scavenging, reducing and chelating activities of flavonol—quercetin were greater than that of flavanols—(+)-catechin and (−)-epicatechin. This can be explained by the presence of 3-hydroxy-4-keto conformation together with a 2,3-double bond in quercetin structure, which ensures extensive electron delocalization and higher stability to the aroxyl radical [12,39]. This structural fragment is associated particularly with a substantial ferrous ions chelation [10], consequently, quercetin can be used as a chelating standard [40]. The highest chelating activity of quercetin observed in our work supports this approach.

Determination of antioxidant activity of optical isomers showed that stereoisomery in flavanols concerning the position of the 3-hydroxyl group was not significantly important for reducing activity and ferrous ions chelation. This is in accordance with the findings reported earlier [10,31].

Greater reducing and chelating activity of chlorogenic and cryptochlorogenic acid compared with that of ferulic acid indicate that esterification of phenolic acids and the additional hydroxyl group on the aromatic ring have the significant influence on the antioxidant activity. These results are consistent with those of Natella et al. [37] and Sova [41], who reported that the antioxidant activity of phenolic acids is enhanced by the introduction of a second hydroxyl group. Chen et al. proved that esters can be an even more effective than free phenolic acids, suggesting that ester functional group favorably stabilize the specific radical species, and thus enhance the radical scavenging activity [42]. In addition, results from our study revealed that the 3-hydroxyl site on the quinic acid ring is more important for reducing activity than the 4-hydroxyl site.

The majority of the tested compounds in the ABTS and FRAP assays possessed higher free radical scavenging and reducing activities than Trolox (TEAC_ABTS_ > 1 and TEAC_FRAP_ > 1, respectively). Scientific studies confirm that natural phenolic compounds have similar or even greater antioxidant activity by comparison with Trolox [9,38,43]. However, all tested compounds in FIC assay had lower chelating activity than EDTA. This suggests that the chelating activity of phenolic compounds is slightly weaker than free radical scavenging and reducing activities.

The overall results of antioxidant activity of tested phenolic compounds indicate that procyanidins C1 and A2 are the major contributors to the antioxidant properties of lingonberry leaves extracts. This explains why extracts from ‘Kostromskaja rozovaja’, ‘Rubin’ cultivars, and *V. vitis-idaea* var. *leucocarpum*, containing the high procyanidins A2 and C1 content, possessed high reducing and chelating activities. Definitely, the antioxidant activity of the latter cultivar was predetermined by the high contents of flavonol glycosides and hydroxycinnamic acids.

Notwithstanding the fact that arbutin is more related for other biological properties, such as a diuretic, urinary antiseptic, melanin biosynthesis in human skin suppressing [6,18,20], and weak radical scavenging and reducing activities were obtained in the present study, correlation analysis still showed that this compound is related to antioxidant properties of lingonberries. So we assume that extraordinarily high contents of arbutin in all tested cultivars certainly contributes to total antioxidant activity.

One of the weakest antioxidant activity of ‘Erntedank’ cultivar can be explained by low contents of all detected phenolics, especially by small amounts of procyanidins A2 and C1. Low amounts of phenolics were also detected in extracts of ‘Erntesegen’ cultivar, however, interestingly great antioxidant activity, especially radical scavenging properties, were found for this cultivar. We hypothesize that this might be due to the presence of some other compounds and non-phenolic antioxidants, such as organic acids or triterpenoids, that can be found in lingonberry leaves [2,18,44]. Therefore, further research of lingonberry leaves composition is required.

## 4. Materials and Methods

### 4.1. Plant Materials

The lingonberry plant material was collected in the field collection of Botanical Garden of Siauliai University (55° 55′ 57″ N, 23° 16′ 59″ E. (WGS)).

1 subspecies, 1 variety and 10 cultivars of *V. vitis-idaea* were analyzed in the paper.

Subspecies (subsp.) is intraspecific taxa between species and variety (var.) and variety is intraspecific taxa between subspecies and form (f.). First cultivars of *V. vitis-idaea* were the morphotypes selected from natural habitats. Morphotype is clones of lingonberry, which differ morphologically but are not investigated genetically [3].

Characteristic of cultivars. German cultivars: 1) ‘Erntekrone’ (registered in 1978); 2) ‘Erntesegen’ (1981); ‘Erntedank’ (1975). All cultivars as morphotypes were selected from the natural populations. Dutch cultivar: 4) ‘Koralle’ (1969); Swedish cultivars: 5) ‘Sanna’ (1987); 6) ‘Sussi’ (1985). Both cultivars were registered in Denmark and selected from the seeds collected in Smaland (Sweden). Polish cultivar: 7) ‘Masovia’ (1985). Cultivar selected from the natural population in Lasy Bolimowskie forest 60 km from Warsaw. Russian cultivars: 8) ‘Kostromskaja rozovaja’ (1995); 9) ‘Kostromička’ (1995); and 10) ‘Rubin’ (1997). All cultivars selected from wild plants [3].

Characteristic of subspecies. *V. vitis-idaea* subsp. *minus* (Lodd.) Hult. is spread in arctic and subarctic zones of Eurasia and North America.

Characteristic of variety. *V. vitis-idaea* var. *leucocarpum* Asch. et Magnus. In 1993 the population belonging to this variety (plants with white berries) was found in the forest of Svencioneliai district, Lithuania [45]. This variety is included to The List of National Genetic Resources of Lithuania Republic.

In 2006 *V. vitis-idaea* subsp. *minus* was received as seeds from The Montreal Botanical Garden (Canada). In 2005 *V. vitis-idaea* var. *leucocarpum* and all cultivars, except ‘Koralle’ were received from the Institute of Botany of the Nature Research Centre (Vilnius) as clones. In 2004 clones of ‘Koralle’ were obtained from Vilnius University Botanical Garden.

Specimens of cultivars and lower taxa of lingonberries are deposited in the Herbarium of the Botanical Garden of Siauliai University (HUS).

**Figure A1:**
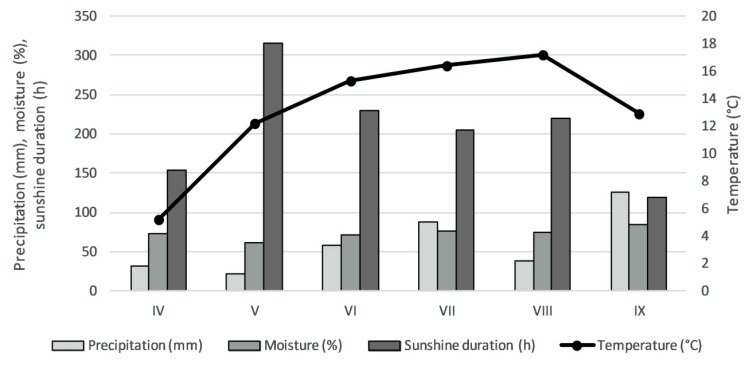
Cultivation conditions of tested lingonberries (dynamics of meteorological factors).

Cultivation conditions. 1. The meteorological data (temperature (°C); precipitation (mm); moisture (%) and sunshine duration (h)) were obtained from the archive of Lithuanian Hydrometeorological Service under the Ministry of Environment. Dynamics of meteorological factors are presented in Appendix A (Figure A1). 2. Plants were cultivated in a medium-sunny place, in acid peaty and well-drained soil. Fertilization and irrigation were applied during the season according to the meteorological situation. Plant raw material for research was collected in September 2017 and dried in 40 °C.

### 4.2. Chemicals and Solvents

Distilled water was purified using a Milli–Q system (Millipore, Bedford, MA, USA). Ethanol (96%) was obtained from Vilniaus degtine (Vilnius, Lithuania). Anhydrous acetic acid (99.8%), hydrochloric acid (37%) were purchased from Sigma–Aldrich (Buchs, Switzerland). The following reagents were used: 2,2′-azino-bis(3-ethylbenzothiazoline-6-sulfonic acid) diammonium salt (ABTS), 2,4,6-Tri-(2-pyridyl)-S-triazine (TPTZ), ferric chloride hexahydrate (FeCl_3_ × 6 H_2_O), sodium acetate (CH_3_COONa), 3-(2-pyridyl)-5,6-bis-(4-phenyl-sulfonic acid)-1,2,4-triazine (Ferrozine), obtained from Sigma-Aldrich (Buchs, Switzerland); potassium persulfate (K_2_S_2_O_8_), anhydrous ferrous chloride (FeCl_2_) from Alfa Aesar (Karlsruhe, Germany). The following standards were used: 6-hydroxy-2,5,7,8-tetramethylchroman-2-carboxylic acid (Trolox), ethylene diamine tetra acetic acid (EDTA), quercetin, chlorogenic acid, cryptochlorogenic acid, ferulic acid, procyanidin A2 from Sigma–Aldrich (Buchs, Switzerland); arbutin, (+)-catechin, (−)-epicatechin from Fluka (Buchs, Switzerland); hyperoside, astragalin, rutin, isorhamnetin-3-*O*-glucoside, quercitrin from Extrasynthese (Genay, France); procyanidin C1, avicularin from ChromaDex (Irvine, CA, USA). All the reagents and standards were of analytical grade.

### 4.3. Sample Preparation

#### 4.3.1. Preparation of the Lingonberry Leaves Extracts

The lingonberry leaves extracts were prepared using 0.25 g of dried raw material and 25 mL of 60% ethanol (1:100). The samples were extracted in an ultrasonic bath (Elmasonic P, Singen, Germany) at 25 °C for 15 min. The extracts were filtered through a 0.22 μm pore size filter (Carl Roth GmbH, Karlsruhe, Germany).

#### 4.3.2. Preparation of the Phenolic Compounds

The stock solutions of phenolic compounds were prepared in 96% ethanol. For the ABTS and FRAP assays, the stock solutions were diluted to five concentrations (concentration ranges shown in Table 3 and Table 4). For the FIC assay, all stock solutions were diluted to final 20 µg/mL concentration.

### 4.4. Determination of Antioxidant Activity

Antioxidant activity of the lingonberry leaves extracts and standards of phenolic compounds, which are most commonly found in lingonberry leaves, was analyzed by spectrophotometric ABTS, FRAP, and FIC assays.

#### 4.4.1. ABTS Radical Cation Decolorization Assay

The radical scavenging activity was evaluated by the scavenging of ABTS radical cation as described by Re et al. with some modifications [46]. ABTS aqueous solution was mixed with K_2_S_2_O_8_ to obtain a concentration of 2 mM. The stock solution was left for 16 h in the dark at room temperature. The working solution was then prepared by diluting the stock solution with water to obtain an absorbance of 1.0 at 734 nm against the blank (water). A volume of 20 μL of each extract (diluted 20 fold) or phenolic compound (at various concentration) was mixed with 3 mL of ABTS working solution. After 1 h, the decrease of absorbance was measured at 734 nm.

ABTS radical cation scavenging activity of extracts was expressed as antioxidant Trolox equivalents (TE) per gram of material. Calculated as follows:

(1) $${\mathrm{TE}}_{\mathrm{ABTS}}=\frac{c\times V}{m},\;\mathrm{mg}/g\;\mathrm{DW}$$

where: c—Trolox concentration in mg/mL from the calibration curve; V—the volume in mL; m—the exact weight of the dry material, g.

The obtained absorption decrease of five different concentrations of tested phenolic compounds and Trolox were used to construct calibration curves (Table 3). Scavenging activity of the tested phenolic compounds was expressed according to Koleva et al. as TEAC_ABTS_ [47], and calculated using the following equation:

(2) $${\mathrm{TEAC}}_{\mathrm{ABTS}}=\frac{a_{\mathrm{sample}}}{a_{\mathrm{trolox}}}$$

where: a_sample_—the slope of the sample obtained from the calibration curve by the ABTS assay; a_trolox_—the slope of Trolox obtained from the calibration curve by the ABTS assay.

#### 4.4.2. Ferric Reducing Antioxidant Power (FRAP) Assay

The ability to reduce ferric ions was measured using a modified version of the method described by Benzie and Strain [48]. Briefly, 20 μL of each extract (diluted 20 folds) or phenolic compound (at various concentration) was added to 3 mL of freshly prepared working FRAP reagent consisted of a 10:1:1 ratio of solutions: 300 mM of acetate buffer (pH 3.6), 10 mM TPTZ dissolved in 40 mM HCl, and 20 mM FeCl_3_ × 6 H_2_O. The absorbance of the mixture was measured at 593 after 1 h incubation at room temperature. The sample substituted by solvent was used as a blank.

Ability to reduce ferric ions by the extracts was expressed as antioxidant Trolox equivalents (TE) per gram of material. Calculated as follows:

(3) $${\mathrm{TE}}_{\mathrm{FRAP}}=\frac{c\times V}{m},\;\mathrm{mg}/g\;\mathrm{DW}$$

where: c—Trolox concentration in mg/mL from the calibration curve; V—the volume in mL; m—the exact weight of the dry material, g.

The obtained absorption of five different concentrations of tested phenolic compounds and Trolox were used to construct calibration curves (Table 4). Reducing activity of the tested phenolic compounds was expressed as TEAC_FRAP_ [47], and calculated using the following equation:

(4) $${\mathrm{TEAC}}_{\mathrm{FRAP}}=\frac{a_{\mathrm{sample}}}{a_{\mathrm{trolox}}}$$

where: a_sample_—the slope of the sample obtained from the calibration curve by the FRAP assay; a_trolox_—the slope of Trolox obtained from the calibration curve by the FRAP assay.

#### 4.4.3. Ferrous Ions Chelating (FIC) Activity Assay

The chelation of ferrous ions was evaluated by the method according to Ye et al. [49], Zengin and Aktumsek [50] with slight modification. A volume of 1 mL of sample solution (extract diluted 3 fold or 20 µg/mL of the phenolic compound) was mixed with 50 µL of 2 mM FeCl_2_ solution. After 5 min, the reaction was initiated by the addition of 0.2 mL of 5 mM ferrozine solution. The mixture was shaken and allowed to react at room temperature for 10 min. The absorbance of the solution was thereafter measured at 562 nm against the blank. To correct for background absorbance, a blank was prepared for each measurement by replacing ferrozine to solvent. The sample substituted by solvent was used as a negative control, and strong chelating agent—EDTA in a range 0–320 μg/mL was used as a positive control. The ability of the sample to chelate ferrous ions was calculated using the following equation:

(5) $$\mathrm{Ferrous}\;\mathrm{ions}\;\mathrm{chelating}\;\mathrm{activity}\;(\%)\;=\left(1-\frac{\mathrm{As}}{\mathrm{Ac}}\right)\times 100\%$$

where: A_s_—absorbance in the presence of the sample; A_c_—absorbance of negative control.

### 4.5. Qualitative and Quantitative Analysis by HPLC–PDA Method

HPLC analysis was performed using a “Waters e2695 Alliance system” (Waters, Milford, MA, USA) with a photodiode array detector “Waters 2998” according to the HPLC–PDA method for phenolic compounds reported by Raudone et al. [51]. Briefly, the“ACE”(ACT, UK) column (C18,150 mm × 4.6 mm, particle size 3 μm) column was used. The gradient consisted of eluent A (0.05% trifluoracetic acid) and B (acetonitrile) and followed: 0–5 min–12% B, 5–50 min–12–30% B, 50–51 min–30–90% B, 51–56 min–90% B, and 57 min–12% B with the flow rate–0.5 mL/min and injection volume–10μL. The analyte and reference compound retention time and UV absorption spectra were used for peak identification.

### 4.6. Statistical Analysis

Statistical analysis was performed using SPSS 21.0 (SPSS Inc., Chicago, IL, USA) and Microsoft Office Excel 2010 (Microsoft, Redmond, WA, USA). All measurements were made in triplicate, and results were expressed as mean ± standard deviation (SD). Simple linear regression analysis was performed to calculate the concentration-response relationship of each investigated compound by ABTS, FRAP and FIC assays. Correlations were tested by using the Pearson correlation test. The hierarchical cluster analysis using Euclidean distances was performed. One–way analysis of variance was performed by ANOVA test. Significant differences between means were determined by Tukey HSD multiple comparison test. The *p*-values less than 0.05 were considered statistically significant.

## 5. Conclusions

Lingonberry leaves could be a promising source of bioactive compounds with notable antioxidant activity. The determined fingerprint profiles are important tools to prove the authenticity of lingonberry products. Therefore, the identification of secondary metabolites could be very important in chemotaxonomical and phytogeographical aspect. ‘Kostromskaja rozovaja’, ‘Rubin’ cultivars and *V. vitis-idaea* var. *leucocarpum*, possessed the greatest reducing, radical scavenging and chelating activities. Procyanidins A2 and C1 could be proposed as markers of radical scavenging, reducing and chelating activities of lingonberries.

## Figures and Tables

**Table 1:** Contents of flavonols and simple phenols (µg/g DW) of Lingonberry leaves extracts.

Cultivars	Compounds				
	Astragalin	Avicularin	Hyperoside	Quercitrin	Arbutin
‘Sussi’	53.22 ± 2.17 d ^1^	1681.92 ± 68.66 a	1744.85 ± 71.23 a	2686.48 ± 109.68 a,b	16,445.51 ± 671.39 a,b
‘Erntekrone’	5.19 ± 0.21 a	3479.62 ± 142.06 e	4380.64 ± 178.84 e	1692.27 ± 69.09 d	12,441.17 ± 507.91 a,c
‘Masovia’	3.07 ± 0.13 a	2313.59 ± 94.45 b	2522.88 ± 103.00 b	1676.31 ± 68.43 d	20,997.03 ± 857.20 b,d
‘Koralle’	6.53 ± 0.27 e	2261.69 ± 92.33 b,c	2939.20 ± 119.99 c	7826.68 ± 319.52 f	13,755.55 ± 561.57 a
*V. vitis-idaea* subsp. *minus*	8.89 ± 0.36 a	2419.27 ± 98.77 b,d	5771.34 ± 235.61 d	5782.61 ± 236.07 g	28,708.70 ± 1172.03 e
‘Kostromskaja rozovaja’	114.67 ± 4.68 b,c	2158.17 ± 88.11 b,c	2342.03 ± 95.61 b	1871.89 ± 76.42 d,e	56,968.56 ± 2325.73 g
‘Sanna’	4.38 ± 0.18 a	2179.45 ± 88.98 f	1926.56 ± 78.65 a	2632.32 ± 107.46 a,b	7070.25 ± 288.64 c
‘Rubin’	120.60 ± 4.92 b	3113.46 ± 127.11 g	3084.12 ± 125.91 c	2628.62 ± 107.31 a,b	36,059.47 ± 1472.12 f
‘Kostromička’	108.79 ± 4.44 c	2715.82 ± 110.87 d	3520.41 ± 143.72 f	2471.29 ± 100.89 a,c	29,877.25 ± 1219.73 e,f
*V. vitis-idaea* var. *leucocarpum*	82.02 ± 3.35 f	5503.44 ± 224.68 g	5682.61 ± 231.99 d	2139.10 ± 87.33 c,e	23,356.78 ± 953.54 d,e
‘Erntesegen’	23.15 ± 0.95 g	1941.79 ± 79.27 c	1764.92 ± 72.05 a	1723.17 ± 70.35 d,e	8963.10 ± 365.92 c
‘Erntedank’	111.77 ± 4.56 c	1946.56 ± 79.47 c	1561.43 ± 63.74 a	2953.06 ± 120.56 b	7443.27 ± 303.87 c

^1^ Contents marked without the same letters (a, b, c, d, e, f, g) in the columns indicate statistically significant differences among cultivars or lower taxa (*p* < 0.05).

**Table 2:** Contents of flavanols, proanthocyanidins, and hydroxycynnamic acids (µg/g DW) of Lingonberry leaves extracts.

Cultivars	Compounds					
	(+)-Catechin	(−)-Epicatechin	Procyanidin C1	Procyanidin A2	Chlorogenic Acid	Cryptochlorogenic Acid
‘Sussi’	897.82 ± 36.65 a ^1^	252.14 ± 10.29 c	1332.85 ± 54.41 a,b	1040.11 ± 42.46 a	346.25 ± 14.14 a	417.74 ± 17.05 b
‘Erntekrone’	595.09 ± 24.29 b	26.27 ± 1.07 a	1071.21 ± 43.73 a,c	3609.57 ± 147.36 b	318.35 ± 13.00 a	368.86 ± 15.06 b,c
‘Masovia’	2954.64 ± 120.62 e	571.32 ± 23.32 d	1090.83 ± 44.53 a,c	3554.68 ± 145.12 b	532.09 ± 21.72 b	334.89 ± 13.67 c
‘Koralle’	605.81 ± 24.73 b,c	71.80 ± 2.93 b	1248.83 ± 50.98 a,b,d	3003.60 ± 122.62 c,d	189.75 ± 7.75 d	217.17 ± 8.87 a
*V. vitis-idaea* subsp. minus	247.92 ± 10.12 d	34.29 ± 1.40 a,b	1310.87 ± 53.52 a,d	7074.60 ± 288.82 g	182.31 ± 7.44 d	673.22 ± 27.48 d
‘Kostromskaja rozovaja’	2273.92 ± 92.83 f	743.67 ± 30.36 e	2481.71 ± 101.32 e	3027.88 ± 123.61 c,f	486.65 ± 19.87 b,c	942.07 ± 38.46 f
‘Sanna’	141.29 ± 5.77 d	60.48 ± 2.47 a,b	1431.05 ± 58.42 b	1364.89 ± 55.72 a	442.12 ± 18.05 c	184.24 ± 7.52 a
‘Rubin’	2093.02 ± 85.45 g	669.11 ± 27.32 f	2525.23 ± 103.09 e	3109.55 ± 126.95 d,e,f	175.47 ± 7.16 d	760.37 ± 31.04 e
‘Kostromička’	767.38 ± 31.33 a,c	33.11 ± 1.35 a,b	3894.92 ± 159.01 f	2684.38 ± 109.59 c	444.92 ± 18.16 c	704.39 ± 28.76 d,e
*V. vitis-idaea* var. *leucocarpum*	1705.80 ± 69.64 h	131.83 ± 5.38 g	4645.97 ± 189.67 g	3448.43 ± 140.78 b,e	954.03 ± 38.95 e	1025.34 ± 41.86 g
‘Erntesegen’	168.93 ± 6.90 d	n.d. ^2^	1055.40 ± 43.09 c,d	1365.75 ± 55.76 a	243.70 ± 9.95 f	218.81 ± 8.93 a
‘Erntedank’	171.29 ± 6.99 d	n.d.	936.48 ± 38.23 c	476.93 ± 19.47 h	352.68 ± 14.40 a	231.42 ± 9.45 a

^1^ Contents marked without the same letters (a, b, c, d, e, f, g, h) in the columns indicate statistically significant differences among cultivars or lower taxa (*p* < 0.05); ^2^ n.d.—peak not detected.

**Table 3:** Calibration curves (y = ax + b) data obtained by ABTS assay for tested compounds.

Compound	Concentration Range (mM)	Slope (a)	Intercept (b)	Correlation (R)	Coefficient of Determination (R^2^)
Trolox	0.13–2.00	0.3559	+0.0580	0.9983	0.9966
Quercetin	0.10–1.66	0.6709	−0,0028	0.9968	0.9935
Quercitrin	0.06–0.89	0.3810	+0.0538	0.9986	0.9973
Rutin	0.05–0.82	0.5378	+0.0627	0.9952	0.9976
Avicularin	0.07–1.15	0.5378	+0.1791	0.9988	0.9977
Hyperoside	0.05–0.86	0.5861	+0.0224	0.9975	0.9981
Isorhamnetin-3-*O*-glucoside	0.07–1.05	0.3332	+0.0642	0.9986	0.9993
Astragalin	0.07–1.12	0.4714	+0.0989	0.9911	0.9955
(+)-Catechin	0.09–1.38	0.5544	+0.0632	0.9964	0.9929
(−)-Epicatechin	0.09–1.38	0.6069	+0.0649	0.9969	0.9938
Procyanidin C1	0.04–0.58	1.0404	+0.9958	0.9958	0.9917
Procyanidin A2	0.05–0.87	0.7511	+0.1408	0.9995	0.9990
Arbutin	0.11–1.84	0.3392	+0.0512	0.9960	0.9920
Ferulic acid	0.16–2.58	0.2730	+0.1183	0.9966	0.9932
Chlorogenic acid	0.09–1.41	0.3038	+0.0775	0.9975	0.9950
Cryptochlorogenic acid	0.02–0.28	0.2761	+0.0151	0.9969	0.9938

**Table 4:** Calibration curves (y = ax + b) data obtained by ferric reducing antioxidant power (FRAP) assay for tested compounds.

Compound	Concentration Range (mM)	Slope (a)	Intercept (b)	Correlation (R)	Coefficient of Determination (R^2^)
Trolox	0.13–2.00	0.5277	−0.0165	0.9998	0.9997
Quercetin	0.10–1.66	0.9283	−0.0671	0.9996	0.9992
Quercitrin	0.06–0.89	0.5712	−0.0217	0.9975	0.9950
Rutin	0.05–0.82	0.7531	−0.0157	0.9969	0.9938
Avicularin	0.07–1.15	0.5936	+0.0268	0.9950	0.9900
Hyperoside	0.05–0.86	0.6692	−0.0121	0.9995	0.9989
Isorhamnetin-3-*O*-glucoside	0.07–1.05	0.2559	+0.0292	0.9951	0.9903
Astragalin	0.07–1.12	0.3119	−0.0231	0.9976	0,9951
(+)-Catechin	0.09–1.38	0.5836	−0.0203	0.9980	0.9960
(−)-Epicatechin	0.09–1.38	0.6172	−0.0080	0.9991	0.9981
Procyanidin C1	0.04–0.58	1.5679	+0.0101	0.9999	0.9998
Procyanidin A2	0.05–0.87	1.0766	−0.0248	0.9960	0.9921
Arbutin	0.11–1.84	0.1826	−0.0021	0.9957	0.9957
Ferulic acid	0.16–2.58	0.3173	+0.0708	0.9961	0.9914
Chlorogenic acid	0.09–1.41	0.5937	+0.0218	0.9992	0.9984
Cryptochlorogenic acid	0.02–0.28	0.4563	+0.0092	0.9972	0.9944
